# Acute Severe Seronegative Autoimmune Hepatitis With Undiagnosed Graves’ Disease

**DOI:** 10.7759/cureus.26884

**Published:** 2022-07-15

**Authors:** Mohammad B Memon, Patrick Duplan, Sindu Iska, Erik Slabaugh, JigneshKumar Patel, Shaival Thakore

**Affiliations:** 1 Graduate Medical Education, HCA Florida Bayonet Point Hospital, Hudson, USA; 2 Internal Medicine, HCA Florida Bayonet Point Hospital, Hudson, USA; 3 Gastroenterology, HCA Florida Bayonet Point Hospital, Hudson, USA

**Keywords:** graves’ disease, acute fulminant hepatitis, acute fulminant hepatitis with parenchymal collapse, seronegative autoimmune hepatitis, jaundice, transaminitis, seronegative, autoimmune hepatitis

## Abstract

Autoimmune hepatitis (AIH) is a progressive inflammatory condition hypothesized to be a T-lymphocyte (T-cell)-mediated immune response that commonly affects females more than males. Given its proposed mechanism associated with immune response, it is more likely to present with other autoimmune conditions, particularly autoimmune disorders associated with the thyroid. AIH can be difficult to diagnose as it is a diagnosis of exclusion. AIH lacks validated algorithms for proper diagnosis and can seldom present with negative antibodies. If not fully worked up, AIH may progress to cirrhosis and even increase the risk of malignancy. Therefore, a liver biopsy is a crucial step in the workup for AIH. We report a rare case of acute severe AIH associated with negative antibodies and undiagnosed Graves’ disease.

## Introduction

According to the National Institute of Allergy and Infectious diseases, more than 80 autoimmune diseases are currently known. This correlates to roughly 4% of the world’s population being affected by one of the autoimmune diseases [1]. Autoimmune hepatitis (AIH) is commonly defined as a chronic inflammatory cascade condition secondary to immune-mediated responses. Such responses encompass circulating autoantibodies and hypergammaglobulinemia which ultimately lead to fibrotic and necroinflammatory changes in the liver. These immune-mediated responses show a strong response to corticosteroids and/or immunosuppressive therapies [2,3]. Additionally, AIH is associated with other autoimmune diseases such as inflammatory bowel disease, autoimmune thyroiditis, and even rheumatoid arthritis [2,4].

There are three major types of AIH: Type I, Type II, and other categories. Type I is usually characterized by the presence of anti-smooth muscle antibodies (ASMA), antinuclear antibodies (ANA), or anti-soluble liver antigen antibodies (SLA). Type I is most commonly associated with autoimmune thyroid disease, rheumatoid arthritis, inflammatory bowel diseases, or overlaps with atypical perinuclear staining antineutrophil cytoplasmic antibodies (50-92%) [2,3]. Type II is primarily seen in patients less than 14 years of age and is associated with liver/kidney microsome type 1 (most common) or type 3 (anti-LKM1 or anti-LKM3), liver cytosol type 1 (anti-LC1). Other types can be defined as graft-dysfunction-mimicking AIH seen in patients receiving liver transplants or include groups that resemble AIH, which overlap other conditions such as primary biliary cholangitis [5].

The incidence and prevalence of AIH vary between continents and even between countries. One of the challenges of obtaining accurate incidence and prevalence is the lack of a signature diagnostic marker. AIH can be suspected after ruling out viral etiologies, drug-induced hepatotoxicity, liver diseases, and genetic conditions such as Wilson’s disease and hereditary hemochromatosis. As of 2021, New Zealand reported an incidence of 1.93 per 100,000 persons. A literature review estimated AIH yearly incidence in America to be 1 per 100,000 persons as of April 2019 [6]. The overall prevalence of AIH is 3 per 100,000 in the adult population [2].

Clinical presentation of AIH varies from asymptomatic, chronic, subacute, acute, and even life-threatening fulminant disease. The asymptomatic presentation of AIH is seen in up to 34% of patients, where liver enzymes scarcely improve over the course of the disease. The chronic nonspecific presentation includes symptoms such as fatigue, which is the most common, followed by malaise, arthralgia, or amenorrhea. AIH does not usually present with physical examination findings; however, jaundice or signs of cirrhosis can be present.

The asymptomatic or chronic clinical presentation of AIH is common in patients with chronic liver disease but can quickly transform into acute AIH. Acute severe AIH is defined as the onset of presentation to be less than 26 weeks, an international normalized ratio (INR) greater than 1.5, and no histologic evidence of cirrhosis [7]. A study showed that the 10-year survival rate is up to 67% in untreated asymptomatic AIH compared to 98% in treated symptomatic severe AIH [2]. AIH is a diagnosis of exclusion and requires a liver biopsy to make a proper diagnosis. This case report describes a patient with acute severe seronegative AIH with concurrent undiagnosed Graves’ disease.

## Case presentation

A 40-year-old female patient with a smoking history of 20 pack-years presented to the emergency department (ED) with chief complaints of scleral icterus, jaundiced skin, and abdominal pain of four-day duration. She reported fatigue, poor appetite, nausea, and decreased oral intake. During the same time frame, she had non-bloody diarrhea and occasional episodes of clay-colored stool. She noted her abdominal pain to be intermittent, not related to any food intake, and non-radiating. She denied any over-the-counter medications to help alleviate her symptoms.

In the ED, the vital signs were remarkable for tachycardia with a heart rate of 111 beats per minute, elevated blood pressure of 148/83 mmHg, and oxygen saturation of 90% on room air. Physical examination was only remarkable for diffuse skin jaundice, scleral icterus, and mild bilateral lower quadrant tenderness on deep palpation. Initial complete blood count (CBC) was only remarkable for a platelet count of 115 × 103/μL. Complete metabolic panel (CMP) was remarkable for total bilirubin of 15.94 mg/d: (0.20-1), direct bilirubin of 13.38 mg/dL (0-0.2), aspartate transaminase (AST) of 1,419 U/L (15-37), alanine transaminase (ALT) of 1,060 U/L (12-78), alkaline phosphatase (ALP) of 149 U/L (45-117), gamma-glutamyl transpeptidase (GGT) of 107 U/L (5-85), and lactate dehydrogenase (LDH) of 354 U/L (100-240). The coagulation panel was remarkable for an international normalized ratio (INR) of 1.6 (0.8-1.1) and prothrombin (PT) of 18.2 seconds (10-12.8) (Table 1).

**Table 1:** Remarkable initial laboratory findings including CBC, CMP, and coagulation panel. CBC: complete blood count; CMP: complete metabolic panel; AST: aspartate transaminase; ALT: alanine transaminase; ALP: alkaline phosphatase; GGT: gamma-glutamyl transpeptidase; LDH: lactate dehydrogenase; INR: international normalized ratio; PT: prothrombin

	Normal range	Initial
Platelet		115
Total bilirubin	0.2–1 mg/dL	15.94
Direct bilirubin	0–0.2 mg/dL	13.38
AST	15–37 U/L	1,419
ALT	12–78 U/L	1,060
ALP	45–117 U/L	149
GGT	5–85 U/L	107
LDH	100–240 U/L	354
INR	0.8–1.1	1.6
PT	10–12.8 seconds	18.2

On admission, computed tomography (CT) scan of the abdomen showed a mildly enlarged spleen, a 5 mm left renal cyst, moderate pelvic ascites, and gallbladder with pericholecystic fluid. Ultrasound of the abdomen showed gallbladder polyps with no ductal dilation. The patient was admitted for management of her acute hepatitis. Further laboratory studies were done to rule out common hepatitis etiologies. Hemoglobin A1c, amylase, lipase, and lipid panel were within normal limits. Ceruloplasmin level was 24.8 mg/dL (19-39). Toxicology was done for opiates, barbiturates, tricyclics, phencyclidine, amphetamine, cocaine, marijuana, and alcohol level.

Her thyroid laboratory findings were as follows: thyroid-stimulating hormone (TSH) of less than 0.01 mIU/mL (0.36-3.74); free thyroxine (FT4) levels over 6.99 ng/dL (day one) (0.7-1.8), 6.35 ng/dL (day two), 5.20 ng/dL (day seven), 4.69 ng/dL (day nine), 3.57 ng/dL (day 56); and total triiodothyronine (T3) greater than 391 ng/dL (86-192) (day one), 244 ng/dL (day two), 339 ng/dL (day seven), 266 ng/dL (day 56) (Table 2). An immunology panel was done to rule out autoimmune causes associated with hepatitis. The immunology panel was remarkable for thyroid-stimulating immunoglobulin (TSI) 17.8 IU/L (0-0.55) and thyroid-stimulating immunoglobulin index (TSII) 14.10 (0-0.55) and 4 on day 56. Thyroid peroxidase was 43 IU/mL (0-34), anti-smooth antibody was 16 units (0-19), anti-mitochondrial antibody was 0.38 Index (0-0.9), ANA screen was negative, and immunoglobulin (Ig)G was 1,218 mg/dL (700-1,600) (Table 3).

**Table 2:** Thyroid panel findings. TSH: thyroid-stimulating hormone; FT4: free thyroxine; T3: total triiodothyronine

	Normal range	Admission	Timeline		
TSH	0.36–3.74 mIU/mL	<0.01			
FT4	0.7–1.8 ng/dL	>6.99	6.35 (day two)	5.20 (day seven)	3.57 (day 56)
T3	86–192 ng/dL	>391	244 (day two)	339 (day seven)	266 (Day 56)

**Table 3:** Immunology panel findings. TSI: thyroid-stimulating immunoglobulin; TSII: thyroid-stimulating immunoglobulin index; ANA: antinuclear antibody; IgG: immunoglobulin G

	Normal Range	Result
TSI	0–0.55 IU/L	17.8
TSII	0–0.55 IU/L	14.1
Thyroid peroxidase	0–34 IU/mL	43
Anti-smooth antibody	0–0.9 Index	0.38
ANA		Negative
IgG	700–1,600 mg/dL	1,218

An infectious panel was done to rule out other causes of hepatitis. Hepatitis A, B, C, D, and E were negative. Human immunodeficiency virus and cytomegalovirus were negative. *Bordetella pertussis* IgM antibody was 4.9 Index (0.0-0.9), *Bordetella pertussis* IgG antibody was 4.52 (0.00-0.94), Epstein-Barr virus (EBV) capsid Ag IgG antibody was over 8 AI (0-0.8), EBV capsid Ag IgM was 0.2 AI (0-0.8), and EBV early antigen was less than 0.20 AI (0-0.8) (Table 4).

**Table 4:** Infectious panel titers. HIV: human immunodeficiency virus; CMV: cytomegalovirus; EBV: Epstein-Barr virus; IgM: immunoglobulin M; IgG: immunoglobulin G; Ag: antigen; Ab: antibody

	Normal Range	Result
Hepatitis A, B, C, D, and E	Negative	
HIV	Negative	
CMV	Negative	
Bordetella pertussis IgM Ab	0–0.9 Index	4.9
Bordetella pertussis IgG	0–0.94 Index	4.52
EBV capsid Ag IgM	0–0.8 AI	0.2
EBV capsid Ag IgG	0–0.8 AI	8
EBV early antigen	0–0.8 AI	0.2

On hospital days one to three, she was evaluated by the gastroenterology service for severe jaundice and persistently elevated transaminase levels. Viral panels and toxicology were negative. Thyroid antibodies were consistent with autoimmune thyroid disease. The cause of her hepatitis continued to be inconclusive. Due to her severe acute hepatitis, a Model for End-Stage Liver Disease (MELD) score was calculated to predict the degree of sickness and how likely she would need a liver transplant if no reversible causes were identified. The patient had a MELD score of 22 which translated to three-month mortality of 19.6%. Gastroenterology service recommended transfer to a tertiary hepatobiliary transplant center for transjugular liver biopsy and possible evaluation for a liver transplant. At the same time, the endocrinology service evaluated her for abnormal thyroid function tests. She did not have any overt signs of thyrotoxicosis before or during admission. Her low TSH levels and elevated TSI and TSII levels were consistent with autoimmune thyroid disease or Graves’ disease. Radioactive iodine uptake (RAIU) was postponed as the patient received an iodine contrast-enhanced imaging study, which would confound RAIU scan results. Because thioamides were contraindicated while in acute liver disease, she was started on propranolol 10 mg three times a day.

**Figure 1:**
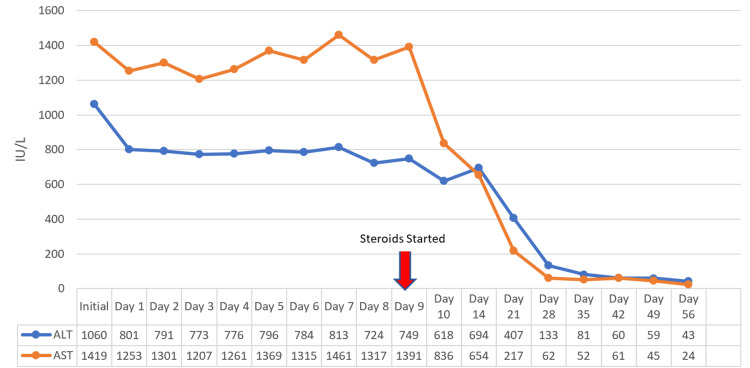
ALT and AST over time. The arrow denotes the day steroids were initiated (day nine). AST: aspartate aminotransferase; ALT: alanine aminotransferase

**Figure 2:**
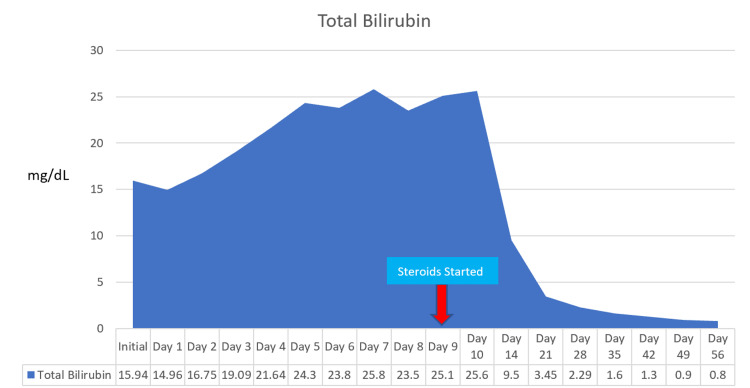
Total bilirubin over time. The arrow indicates the day steroids were initiated (day nine).

On hospital days four to nine, She was transferred to a tertiary care center and evaluated by the transplant service for possible candidacy for a liver transplant. The transplant service recommended a transjugular liver biopsy. Her preliminary pathology report was consistent with AIH. She was started on prednisone 60 mg on day nine, and her liver enzymes improved (Figures 1, 2).

**Figure 3:**
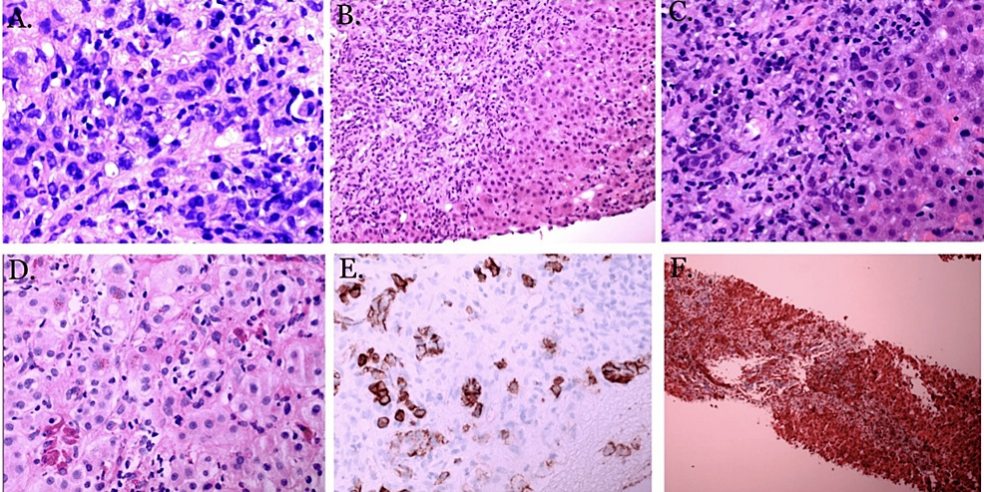
Liver biopsy histology slides. (A-F) Active mixed portal, periportal, and marked lobular hepatitis, marked portal and bridging fibrosis (A-D) associated with bile ductular reaction (F), and CD138 immunostain highlighting portal/septal plasma cells (E).

Pathology was read and confirmed independently by two pathologists. The report of the liver biopsy showed acute fulminant hepatitis with parenchymal collapse. Detailed report of the biopsy showed active mixed portal, periportal, and marked lobular hepatitis, marked portal and bridging fibrosis associated with bile ductular reaction (Figure 3F), CD138 immunostain highlighting portal/septal plasma cells (Figure 3E), CD68 immunostain highlighting Kupffer cells, and some macrophages in the portal/septal space (Figure 3A-3F). All biopsy findings were consistent with the diagnosis of AIH.

## Discussion

Risk factors associated with AIH can be divided into five main types. The five types are gene mutations or predisposing alleles, viral triggers, drug exposure, immune mechanism dysfunction, and individual risk factors [8]. Although the pathogenesis of AIH is not clearly understood, it is believed to be a T-cell-immune-mediated injury. It is worth noting that coexisting autoimmune diseases can be present, with the most common being autoimmune thyroid disease [2]. The risk of AIH actors and the ability to progress are multifactorial. Two cohort studies found 28 out of the 278 AIH patients to have associated autoimmune thyroiditis [9]. Our initial patient presentation was consistent with acute severe AIH; however, throughout her hospital workup, she was also found to have antibodies consistent with Graves’ disease.

Graves’ disease is defined as the most common type of hyperthyroidism. It is an autoimmune condition characterized by autoimmune antibodies against TSH receptors. There is an increase in T4 production and peripheral conversion to T3 in Graves’ disease. The relationship between hyperthyroidism and AIH is not clearly defined, but it is believed that AIH and Graves’ disease are due to T-cell-mediated immune response. A medical literature review found 12 reported cases of AIH with Graves’ disease [10]. A 10-year retrospective cohort study in 146 patients with Graves’ disease evaluated the association of Graves’ disease with abnormal liver enzymes. The most common abnormality was elevated GGT levels compared to ALP levels in other studies [11]. However, this study has a major limitation as some side effects of hyperthyroidism medications could induce liver injury and cause elevated liver enzymes. Severe cholestatic hepatitis due to Graves’ disease is uncommon as it is commonly related to antithyroid medications or thyroid storm. Cholestatic hepatitis is defined as ALP levels greater than three times the upper limit with mild elevation in transaminases [12]. Based on our patient’s ALP, AST, ALT, bilirubin, imagings, immunology panel workup, and liver biopsy findings, our patient’s undiagnosed Graves’ disease was more of an association than causation of her acute severe seronegative AIH.

One of the mechanisms of immune-mediated injury is the malfunction of CD4+CD127-T-cells that contributes to the dysfunction of regulatory T-cells (Treg) in AIH patients. Dysfunction of Treg cells causes the proliferation of CD4+ and CD8+. CD4+T-cells or helper cells are in charge of B-cell antibody production and affect the cytotoxicity of CD8+T-cells. CD4+ is responsible for activating plasma cells usually present in the spleen or peripheral lymph node tissues. There is a possibility of tertiary lymphoid tissue in the intrahepatic system, defined as portal-associated lymphoid tissue, which would produce autoantibodies [8]. Some defining hallmarks of AIH are the presence of autoimmune antibodies and plasma cells on liver biopsy.

An observational study of 86 patients in Italy evaluated the differences between acute AIH and acute viral hepatitis. Acute AIH was more prevalent in females (82%, p < 0.0001) than acute viral hepatitis. Acute AIH had an AST/ALT ratio of about 1.20 (p < 0.0001) compared to acute viral hepatitis with a ratio of 0.61 (p < 0.0001) [13]. Our female patient had an initial AST/ALT ratio of 1.34. As mentioned above, the diagnosis of AIH requires the exclusion of common hepatitis conditions such as infections, drug-induced, and even genetic conditions such as Wilson’s disease. ANA, SMA, anti-LKM, serum globulin, and IgG markers should be part of the primary workup; however, the diagnosis cannot be made until histological findings from a liver biopsy are consistent with AIH [2].

A study showed that in North American patients with acute severe AIH, ANA levels could be negative, and serum IgG levels can be normal in up to 39% of patients, as seen in our patient [2]. It is essential to include the diagnostic scoring system of the International Autoimmune Hepatitis Group that was created in 1993, revised in 1999, and simplified in 2008. Each scoring system has its strength. The original scoring system has greater sensitivity (100%), the revised system is better for atypical cases like our patient, and the simplified scoring system has superior specificity (90%) [2]. A retrospective study conducted between 2011 and 2015 in 101 patients with AIH found that acute severe AIH tends to be more prevalent in seronegative patients (50%) compared to seropositive AIH patients (20.27%) [14]. Our patient’s revised original score for AIH was 16 points, which suggested a definite AIH diagnosis, and a corticosteroid trial was recommended. The AIH scoring system can aid clinicians in making the diagnosis, but due to the complexity of the disease, there has not been a validated management algorithm using the AIH scoring system.

Histological findings for AIH need to be differentiated from drug-induced liver injury (DILIL); however, the hallmark of AIH is defined as interfaced hepatitis. Some histological features that favor AIH more than DILI are severe portal inflammation, prominent intra-acinar eosinophils, prominent portal plasma cells, rosette formation, any level of fibrosis, and severe focal necrosis [15]. Our patient’s liver biopsy pathology report was consistent with severe portal inflammation, portal plasma cells, levels of fibrosis, and necrosis. Corticosteroids block the conversion of T4 to T3 in Graves’ disease [10]. Concerning AIH, corticosteroids decrease gene expressions involved in pro-inflammatory cytokine production and stimulate the proliferation of Treg cells, which reduce liver cell injuries and decrease T-cell immune-mediated damages described in the previous section [6]. Our patient was started on prednisone 60 mg on day nine, according to the American Association for the Study of Liver Diseases (AASLD) guideline for acute severe seronegative AIH, with notable improvement in her liver enzymes 24 hours later [6]. There is usually an 80% improvement or reduction in the transaminase levels within the first eight weeks; however, if no improvement in transaminase levels is noted, then the clinician should suspect an alternative diagnosis [2,16]. Forty days after the initiation of prednisone therapy, our patient’s ALT level reduced by 92.12% and AST levels by 96.76%. In the case of our patient, corticosteroid was beneficial for both Graves’ disease and AIH.

Initiation of early treatment, as noted above, leads to a good prognosis with 10-year survival greater than 80% while the failure of treatment can lead to fulminant hepatitis and lead to the risk of hepatocellular carcinoma with a rate of 3.06 per 10,000. In one study, most patients achieved complete remission within three months but required long-term or permanent immunosuppressive therapy with azathioprine [16]. Given the long-term side effect profile of corticosteroids, alternative usage of azathioprine long-term or indefinitely has also yielded positive outcomes with increased patient tolerability.

## Conclusions

Our case report discusses a rare case of acute seronegative AIH with undiagnosed Graves’ disease. The typical presentation of AIH tends to be chronic with mild elevation of AST and ALT levels; however, it is essential to recognize seronegative AIH because of its atypical presentations. If there is a delay in diagnosing AIH, it could increase the risk of liver cirrhosis and even liver cancer. We encourage an emphasis on liver biopsy once the common etiologies are ruled out regardless of antibodies specific to AIH. There is no clear-cut algorithm for AIH, and understanding the strength of each scoring system is vital to making the proper diagnosis. We hope this case report improves the index of suspicion among clinicians for seronegative AIH and further enhances the medical literature pertaining to this subject.
